# Chemical Fingerprint Analysis for Discovering Markers and Identifying *Saussurea involucrata* by HPLC Coupled with OPLS-DA

**DOI:** 10.1155/2020/7560710

**Published:** 2020-09-19

**Authors:** Qingdong Ma, Xiaoxiang Chen, Ke Zhang, Dahong Yao, Lu Yang, Hangyu Wang, Santai Bulemasi, Jian Huang, Jinhui Wang

**Affiliations:** ^1^School of Pharmacy, Xinjiang Medical University, Urumqi 830054, China; ^2^College of Pharmacy, Key Laboratory of Xinjiang Phytomedicine Resource and Utilization, Ministry of Education, Shihezi University, Shihezi 832002, China; ^3^School of Pharmacy, Harbin Medical University, Harbin 150081, China; ^4^Economic Forest Product Quality Inspection and Testing Center of State Forestry Administration, Xinjiang Academy of Forestry, Urumqi 830052, China; ^5^State Forestry Administration of Xinjiang Altai Mountain, Altai 836505, China

## Abstract

The quality control of *Saussurea involucrata* has been greatly improved by macroscopic and microscopic identification and chemical profiling described in Chinese Pharmacopoeia since 2005. However, these methods have their own limitations, e.g., their dependence on personal experience and expertise, and it is a huge challenge to identify closely related species that share similar or identical morphological characteristics and chemical profiles. A novel and generally accepted identification strategy is urgently needed as a complement to regulations for protecting the public health interests. In this work, a comprehensive chromatographic fingerprint method was developed and tested on 43 samples from four haplotypes of *S. involucrata* according to DNA barcoding. Three common patterns consisting of 20, 14, and 7 common peaks were generated by frequency filters of median, upper quartile, and 100%, respectively. Based on two formerly screened patterns, *S. involucrata* can be effectively identified from its five easily confused snow lotus species, including the most closely related plant (*S. orgaadayi*) in the orthogonal partial least-squares discriminant analysis (OPLS-DA) models. The model is supported by good R and Q coefficients. In addition, different haplotypes of *S. involucrata* can be discriminated in the OPLS-DA model using the 20 common peaks. Among them, peaks 9, 11, 16 (zaluzanin C), and 18 (dehydrocostus lactone) have been identified as fingerprint markers of *S. involucrata* via S-plots and VIP values (>1). Additionally, peaks 19 and 20 were identified as linolenic acid and linoleic acid with anti-inflammatory activity, and they were isolated from the herb for the first time. Collectively, the chromatographic fingerprint of *S. involucrata* can be an effective and integrated method for the identification of authentic herbs from adulterant species or related plants, and discrimination of its different haplotypes provides an objective and reliable tool for quality control.

## 1. Introduction

*Saussurea involucrata* (Kar. et. Kir.) Sch. Bip., an important herb for rheumatic and gynecological diseases, has long been prescribed in Uygur medicine and traditional Chinese medicine [1, 2]. In particular, *Saussurea involucrata* is not only taken as a critical raw drug in compound preparations but also used for a single-herb formulation [3, 4]. Additionally, recent studies have confirmed that *Saussurea involucrata* offers a wide spectrum of bioactivities, such as anti-inflammatory [2, 5], antitumor [6], antihypoxic [7], and antifatigue ones [8], which are closely related to the traditional treatments.

*Saussurea involucrata* has been identified by macroscopic and microscopic characteristics, as the former features primarily rely on the shape of stem leaves, texture of bracteal leaves, and the number of calathidia; the latter have several properties, e.g., pollen grains, glandular hairs, and pappus. However, these methods depend on personal experience and expertise. Furthermore, chlorogenic acid and rutin have been used as the indicator compounds in the quality control of *Saussurea involucrata* in Chinese Pharmacopoeia since 2005. However, chlorogenic acid and rutin are not characteristic compounds of *Saussurea involucrata*; they are only two compounds of more than 70 active chemical constituents that have been identified from *Saussurea involucrata* [1], including sesquiterpenes, phenylpropanoids, and flavonoids. Since the efficacy of herbal medicine depends on more than two chemical compounds that work synergistically, there is a pattern-based approach in the quality control of a medicinal plant known as fingerprint analysis, which can reveal all detectable compounds in herbal medicine, and therefore it has been used for determining the authenticity of herbal drugs. Moreover, chlorogenic acid and rutin are major chemical compositions in a variety of herbal medicines widely. For example, the content of chlorogenic acid is as high as 4.0% in *Lonicerae japonicae* Flos [9], and the content of rutin is as high as 22.0% in *Flos Sophorae* Immaturus [10]. The content of chlorogenic acid and rutin in *Saussurea involucrata* is only 0.15%, according to Chinese Pharmacopoeia, and therefore it may be illegally added with extracts rich in chlorogenic acid and rutin to its raw material of herbal medicinal products to comply with the requirement specified in pharmacopeia. Thus, much effort has been made to develop a more reliable authentication technique. Several HPLC fingerprints have been reported for identification in the quality control of *Saussurea involucrata* in which samples for study were from different batches and areas [11–14]. These samples were not identified with DNA barcoding, and therefore they may not cover a total of four haplotypes of *Saussurea involucrata* in our previous study based on ITS2 sequences, and its four haplotypes are consistent with the results of gene sequences recorded in GenBank and the DNA Barcoding System for Identifying Herbal Medicine, since a medicinal plant has multiple haplotypes on the basis of variable sites when being tested by DNA barcoding, and HPLC fingerprinting has been used for assessment of chemical variability in herbal medicine [15, 16]. The chemical profile of herbal drugs was mostly affected by the medicinal plant gene. Thus, it is necessary to investigate the chemical pattern of *Saussurea involucrata* and identify the herb based on its genetic background. However, these methods were based on compounds with aromatic rings, excluding compounds with double bond such as sesquiterpene lactones in *Saussurea involucrata*. In addition, their common peaks were mainly identified as flavonoids, such as quercetin, hispidulin, and jaceosidin [12]. These compounds need to be reassessed as fingerprint markers for the identification of *Saussurea involucrata*. Moreover, *Saussurea involucrata* is frequently imitated by its adulterants, due to their similar morphological appearances and folk name [17]. In fact, counterfeiting of *Saussurea involucrata* has worsened recently, due to the discovery of a closely related plant, *Saussurea orgaadayi* [18]. Therefore, the identification of chemical markers for the authentication of *Saussurea involucrata* is urgently needed.

In this work, a novel and thorough HPLC fingerprint method was developed, consisting of 20 common peaks and depending on four haplotypes of *Saussurea involucrata* based on the DNA barcoding. Moreover, *Saussurea involucrata* can be discerned from adulterants, including the most closely related plant, in the orthogonal partial least-squares discriminant analysis (OPLS-DA) model. Peaks 9, 11, 16 (zaluzanin C), and 18 (dehydrocostus lactone) are candidates for fingerprint markers for quality control of *Saussurea involucrata*. Additionally, linolenic acid and linoleic acid were isolated from *Saussurea involucrata* for the first time; they are peaks 19 and 20.

## 2. Materials and Methods

### 2.1. Chemicals and Reagents

Reference standards of phytochemical compounds (chlorogenic acid, rutin, and dehydrocostus lactone) were obtained from the National Institutes of Food and Drug Control (Beijing, China). Zaluzanin C and 5, 7, 4′-trihydroxy-3′, 8-dimethoxyflavone were homemade from *Saussurea involucrata* and confirmed by NMR. Phosphoric acid (analytical reagent) was purchased from Tianjin Fuyu Fine Chemical Co., Ltd. (Tianjin, China). HPLC grade methanol and acetonitrile were purchased from J. T. Baker (Phillipsburg, USA). Distilled water was purified by a redistilled water system in the laboratory (Shanghai, China).

### 2.2. Plant Materials

Seventy-two samples were collected from the main distribution regions in Xinjiang, Xizang (Tibet), Sichuan, and Gansu of China (Table 1) and authenticated by Professor Ping Yan, College of Life Science, Shihezi University. The herbs were dried in the shade described in Chinese Pharmacopoeia. Meanwhile, 25 samples were also identified by the DNA barcoding method described in Chinese Pharmacopoeia. First, all samples were identified by morphology; the samples with identical morphology were classified into one group and then tested by DNA barcoding. It is worth mentioning that *Saussurea involucrata* is the only species recorded in Chinese Pharmacopoeia, while the others are not (Table 1). The studied samples contained four haplotypes of the official snow lotus herb, and this was consistent with the results of gene sequences recorded in GenBank and the DNA Barcoding System for Identifying Herbal Medicine.

### 2.3. Sample Preparation

In brief, the samples were pulverized for 5 min to powder, and 0.5 g of each powdered sample was placed in a 50 mL conical flask with stopper. To each sample, 10 mL of methanol was added, and the mixture was sonicated (200 W, 59 kHz) for 15 min. Before HPLC analysis, the solution was filtered through a 0.22 *μ*m syringe-driven filter.

### 2.4. Chromatographic Conditions

The chromatographic fingerprint data were obtained on a high-performance liquid chromatography system (Waters 2695, Waters Corporation, Milford, USA), with binary pumps, a column oven, a degasser, an SM7 autosampler, and a 2998 photodiode array detector. The separation was performed on a VP-ODS column (150 mm × 4.6 mm, 5 *μ*m) maintained at 30°C at a flow rate of 1.0 mL/min. The mobile phases consisted of 0.3% phosphoric acid aqueous solution (A) and acetonitrile (B) with the following gradient program: 5%–50% B for 0–25 min, 50%–98% B for 25–40 min, and hold at 98% B for 40–50 min, back to initial conditions at 5% B for 50–60 min, and hold at 5% B for 60–75 min. The injection volume was 10 *μ*L, and the detection wavelength range was from 190 to 400 nm.

### 2.5. Methodology Validation

The test solutions used for verification were prepared according to Section 2.3. The precision was evaluated for five consecutive tests with the same sample. The repeatability was estimated by six independent parallel samples, and the stability was assessed for one sample at room temperature, which was injected at 0, 2.5, 5, 7.5, 10, and 20 h.

### 2.6. Data Analysis

The primary HPLC data were obtained and transformed into AIA files by Empower software (version 2, Waters Corporation, Milford, USA). The files were subsequently imported to ChemPattern software (version 2.1, Chemmind Technology Co., Ltd., Beijing, China) for further analysis. In brief, all the chromatographic peaks were background subtracted, smoothed, integrated, and aligned. Then, common patterns were generated by peak statistics, frequency filter (0% for blank solvent), and Gaussian curve for profile simulation after removing all peak areas of the first three minutes and blank solvent. All samples of *S. involucrata* were chosen for representative samples for common pattern generation. Fingerprint data were copied to MS Excel 2007, where tables were created and exported to the SIMCA software. Chemometric analyses were performed by SIMCA software (13.0, Umetrics, Umeå, Sweden).

Principal component analysis (PCA) is usually the most popular variable reduction unsupervised technique for the exploratory analysis of fingerprints. Orthogonal partial least-squares discriminant analysis (OPLS-DA) also represents an alternative to traditional methods for selecting the most significant metabolites as markers [19, 20]. The fitting parameter (*R*^2^) and the predictive ability parameter (*Q*^2^) were utilized to evaluate the quality of the models. The value of *Q*^2^ is more than 0.4 for a biological model [20]. In addition, the S-plot is used to select biomarkers due to revealing the relation between the predictive score and component. The VIP (variable importance in the projection) score is more than 1 [20].

## 3. Results and Discussion

### 3.1. Optimization of Extraction Conditions

Chemical constituents of herbal medicine were affected by extraction solvents and methods when a medicinal plant was analyzed for chemical profiles. For example, the extraction efficiency of lignin is revealed by the synergistic effect of hydrogen peroxide and ammonia [21, 22] and is affected by pretreatment, such as presoaking, of the tested material [23–25]. Apart from flavonoids, sesquiterpene lactones of *Saussurea involucrata* were focused by their anti-inflammatory activity [26]. For instance, it has been reported that dehydrocostus lactone, as one of sesquiterpene lactones, possesses the ability of inhibiting inflammation and its content is as high as 1.2% in *Saussurea involucrata* [27, 28]. Sesquiterpene lactones were extracted effectively by sonication using 100% MeOH as a solvent [29]. Therefore, ultrasonic extraction and extraction solvents such as dichloromethane, acetone, and methanol were used for optimization of the extraction method in *Saussurea involucrata*. Moreover, the plant contains water, and the determination of water content should be performed before the extraction of active components [30].

The samples of *Saussurea involucrata* were used for optimizing the ultrasonic extraction process, which included extraction solvents (dichloromethane, 95% ethanol, acetone, and methanol), crude drug-solvent ratios (w/v, 20 times, 30 times, and 40 times), and extraction time (15 min, 30 min, and 45 min) via a univariate approach. The results showed that methanol had the highest extraction efficiency. It was also found that the most efficient parameters were a solid-liquid ratio equal to 20 times and extraction time of 15 min.

### 3.2. Wavelength Selection

**Figure 1:**
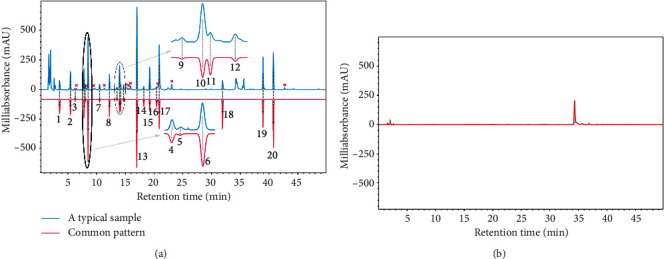
The HPLC chromatograms (203 nm) of a typical sample and common pattern (a) and blank solvent (b).

To select the best wavelength, the characteristic peaks in the fingerprints were investigated at 254 (minor modified), 280, and 330 nm, as previously described in [12, 14, 31]. These wavelengths focus on aromatic compounds. Wavelengths 198 and 203 nm were selected for compounds with double bonds. Although there were some absorption peaks in the blank solvent at 203 nm (Figure 1(b)), the peaks at higher wavelengths were also fully displayed. Additionally, the pattern of the chromatogram at 203 is identical to that at 198 nm, while a lower baseline noise was observed at 203 nm; therefore, the wavelength at 203 nm was selected for analysis.

### 3.3. Methodology Validation

The three parameters for verification, i.e., precision, repeatability, and stability, were evaluated by calculating the relative standard deviation (RSD). The RSD values of the retention time of the 9 main peaks were 0.01%–0.84%, 0.02%–0.22%, and 0.01%–1.13% for precision, repeatability, and stability, respectively, and the RSD values of peak area of the nine main peaks were 0.54%–2.65%, 0.97%–2.72%, and 0.65%–3.57% for precision, repeatability, and stability, respectively (Table 2). The results demonstrate that the established method is reliable and accurate and has good stability.

### 3.4. Chemical Fingerprint Analysis

A total of 20, 14, and seven common peaks were generated by frequency filter with median, upper quartile, and 100% of the chromatographic data of all samples of *Saussurea involucrata* (Figure 1(a) and Table S1). The common pattern of the upper quartile consists of peaks 1–4, 6–8, 10, 11, 15, and 17–20, while the common pattern of 100% of the frequency range contains peaks 4, 6, 8, 15, 17, 19, and 20. Among them, peaks 6, 8, 16, 17, and 18 are chlorogenic acid, rutin, zaluzanin C, 5, 7, 4′-trihydroxy-3′, 8-dimethoxy-flavone, and dehydrocostus lactone, respectively. Peaks 19 and 20 were identified as linolenic acid and linoleic acid, which were isolated from *Saussurea involucrata* for the first time and confirmed by NMR (Fig.  S1–S4). Briefly, the dried aerial parts of *Saussurea involucrata* (5 kg) were extracted with 95% ethanol three times to afford a crude extract, which was suspended in water and partitioned with petroleum ether, dichloromethane, and ethyl acetate to yield a petroleum ether, dichloromethane, ethyl acetate, and aqueous fractions. The mixture (named as SIP) part of petroleum ether and dichloromethane fractions was subjected to silica gel CC eluted with a gradient of petroleum ether-acetone (from 100 : 0 to 0 : 100) to obtain SIP fractions. Fr. SIP–B2 (100 : 1) was chromatographed over an ODS column using a gradient of MeOH–H_2_O (from 50% to 100%) to give Fr. SIP–B2-85-3 (120 mg), which was separated by HPLC, followed by eluting with MeCN–H_2_O (90%), yielding linolenic acid (7.6 mg) and linoleic acid (8.2 mg). Significantly, the two polyunsaturated fatty acids have anti-inflammatory activity [32, 33], showing potential for medicinal applications.

### 3.5. Chemometric Analysis

**Figure 2:**
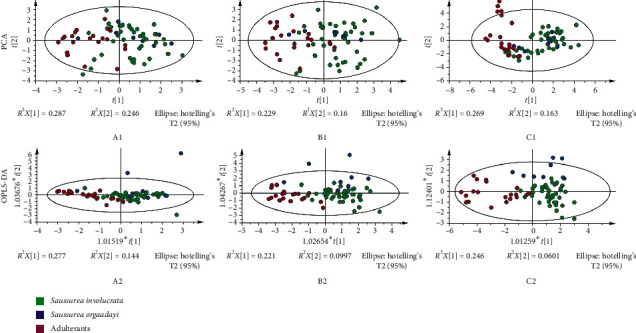
The PCA and OPLS-DA models for classification of *Saussurea involucrata*, *Saussurea orgaadayi*, and adulterants based on chemical fingerprinting. The data were generated by a common pattern from the median (C), upper quartile (B), and 100% of the frequency domain (A).

In herbal fingerprint analysis, principal component analysis (PCA) was commonly the unsupervised applied tool for the exploratory analysis of the reduction. Hence, the common pattern data of 100% of the frequency domain, the upper quartile, and the median, based on the chemical fingerprints of 72 samples, were analyzed by PCA (Figure 2). The results of the score plots (model A1: *R*^2^*X*, 0.533, *Q*^2^, −0.085; B1: *R*^2^*X*, 0.389, *Q*^2^, 0.066; C1: *R*^2^*X*, 0.616, *Q*^2^, 0.149) revealed that the three groups overlapped significantly, which can be attributed to the similarity between related plants. Therefore, the unsupervised method is not suitable for identifying *Saussurea involucrata*.

To explore the underlying differences, OPLS-DA, which is a minimally biased classification technique, was used for selecting. In the constructed models (Figure 2), the model of common pattern with 100% of the frequency domain was still achieved for the unsatisfied *Q*^2^ value (model A2: *R*^2^*X*, 0.421, *R*^2^*Y*, 0.361, *Q*^2^, 0.297). However, two other models were supported by their statistical parameters: models B2 (*R*^2^*X*, 0.321, *R*^2^*Y*, 0.535, *Q*^2^, 0.450) and C2 (*R*^2^*X*, 0.455, *R*^2^*Y*, 0.589, *Q*^2^, 0.434). Among them, C2 was more accurate. Thus, the samples from unknown origin could be screened by the OPLS-DA models.

### 3.6. Identification of *Saussurea involucrata*

**Figure 3:**
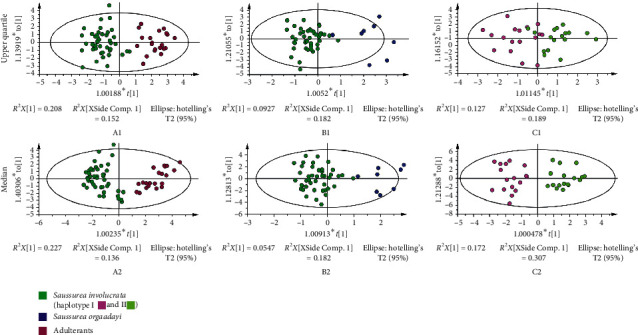
The OPLS-DA models for classification of the *Saussurea involucrata* and adulterants (A), *Saussurea involucrata and Saussurea orgaadayi* (B), and haplotypes I and II of Saussurea involucrata (C). The data were generated using common patterns of the upper quartile (1) and median (2).

Comparing the R and Q coefficients of the OPLS-DA models, the quality of the model between two groups is potentially better than models consisting of three groups. Hence, two groups were constructed by the OPLS-DA models for distinguishing *Saussurea involucrata* from its adulterants or *Saussurea orgaadayi* and discriminating haplotypes I and II of *Saussurea involucrata.* First, the samples from *Saussurea involucrata* and its adulterants were well separated using the common patterns of both the upper quartile (Figure 3 A1) and the median (Figure 3 A2). The median model proved to be more accurate, according to the R and Q values (Table 3). Second, *Saussurea involucrata* and *Saussurea orgaadayi* could be distinguished using the data from the upper quartile (Figure 3 B1) and the median (Figure 3 B2) based on common patterns. Interestingly, the master haplotypes of *Saussurea involucrata* could be separated from one another by the median common pattern model (Figure 3 C2), which indicated that the chemical difference between haplotypes of *Saussurea involucrata* was significant. Therefore, the sample of *Saussurea involucrata* could be identified clearly.

### 3.7. Selection of the Potential Markers

**Figure 4:**
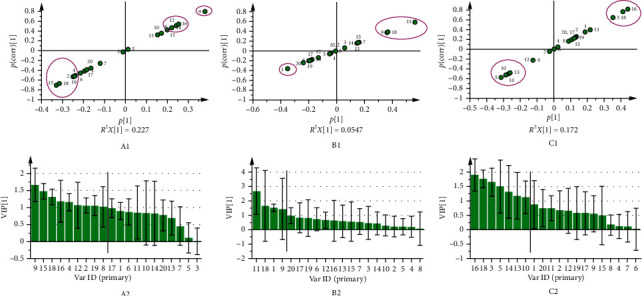
The S plots (1) and VIP (2) for classification of the *Saussurea involucrata* and adulterants (A), *Saussurea involucrata* and *Saussurea orgaadayi* (B), and haplotypes I and II of *Saussurea involucrata* (C). The data were generated using common patterns of the median.

As mentioned previously, the sample separation was based on the chemical fingerprint of the common peaks. Among them, several peaks were identified as potential candidates for markers, due to their high degree of discrimination. S plots and VIP were generated from the 20 common peaks data.  Figure 4 shows that a total of 9 peaks (2, 4, 8, 9, 12, 15, 16, 18, and 19) play a very important role in *Saussurea involucrata* identification. Moreover, peaks 1, 9, 11, and 18 are significant for authenticating *Saussurea involucrata* samples from *Saussurea orgaadayi*. Additionally, 7 peaks (3, 5, 10, 13, 14, 16, and 18) are important compounds for discriminating haplotypes of *Saussurea involucrata*. Collectively, peaks 9, 11, 16, and 18 can be considered as fingerprint markers for *Saussurea involucrata*.

## 4. Conclusion

A comprehensive chemical fingerprint method for the overall quality control of *Saussurea involucrata* has been developed, optimized, and validated. Combined with OPLS-DA, it has been successfully applied to identify *Saussurea involucrata* from its adulterants and the most closely related plant, *Saussurea orgaadayi.* The model is also capable of discriminating its haplotypes. Peaks 9, 11, 16 (zaluzanin C), and 18 (dehydrocostus lactone) can be considered as fingerprint markers for *Saussurea involucrata*. Thus, this provides great insight into the quality control of *Saussurea involucrata,* and this method can be used as a valuable reference in evaluating quality and standardizing the management of herbal medicine.

## Figures and Tables

**Table 1:** Plant materials used in this work.

Species		No.	Origin	Province
*S. involucrata*	I	17	Hejing, Zhaosu, Heshuo, Urumqi, Shihezi, and Bayingolin	Xinjiang
	II	17	Fukang, Jimusa'er, Mulei, Qitai, Hami, Barkol, and Tianchi	Xinjiang
	III	6	Kashgar	Xinjiang
	IV	3	Gongliu	Xinjiang
*S. orgaadayi*		8	Qinghe, Fuyun, Altai, and Keketuohai	Xinjiang
*S. laniceps*		5	Jiulong, Nyingchi, and Nagqu	Sichuan; Xizang
*S. medusa*		6	Qamdo, Tingri, Yadong, Lanzhou, and Ganzi	Xizang; Gansu; Sichuan
*S. tridactyla*		5	Dingri, Xigaze, Nagqu, and Lhasa	Xizang
*S. simpsoniana*		5	Dingri, Xigaze, Yadong, and Lhasa	Xizang
Total		72		

Roman number I is the master haplotype of *S. involucrata*, which possessed another three haplotypes with three variable sites at the 103^rd^ (T-C), 156^th^ (C-T), and 167^th^ (T-C) for IV, III, and II, respectively.

**Table 2:** Method validation.

No.	Precision (RSD, %)		Repeatability (RSD, %)		Stability (RSD, %)	
	Retention time	Peak area	Retention time	Peak area	Retention time	Peak area
1	0.84	1.47	0.22	2.72	1.13	2.38
2	0.65	0.98	0.19	2.46	0.86	1.63
3	0.54	0.54	0.16	1.42	0.68	0.65
4	0.41	1.25	0.13	0.97	0.39	1.06
5	0.36	2.40	0.09	2.60	0.41	1.99
6	0.30	2.34	0.08	2.37	0.28	3.57
7	0.08	1.82	0.03	1.27	0.08	1.48
8	0.01	2.65	0.02	2.10	0.01	2.37
9	0.03	1.42	0.02	2.43	0.02	1.07

**Table 3:** The quality values of *R* and *Q* coefficients.

Common pattern	*Saussurea involucrata* (*n* = 43) vs. adulterants (*n* = 21)				*Saussurea involucrata* (*n* = 43) vs. *Saussurea orgaadayi* (*n* = 8)				Haplotype I (*n* = 17) vs. II (*n* = 17) of *Saussurea involucrata*			
		*R * ^2^ * X*	*R * ^2^ * Y*	*Q * ^2^		*R * ^2^ * X*	*R * ^2^ * Y*	*Q * ^2^		*R * ^2^ * X*	*R * ^2^ * Y*	*Q * ^2^
Upper quartile	A1	0.361	0.843	0.779	B1	0.499	0.766	0.546	C1	0.316	0.586	0.251
Median	A2	0.489	0.849	0.781	B2	0.482	0.772	0.412	C2	0.621	0.870	0.740

## Data Availability

A majority of the data used in this research are included in the article. Other data can be made available upon request from the first author and corresponding author.

## Supplementary Materials

Supplementary Materials The supplementary data consist of Table S1 and Figures S1–S4. Table S1: ratios of the common peak areas from 72 samples with the filter of median, upper quartile, and 100% of the chromatographic data of each sample. Figures S1–S2: ^1^H NMR and ^13^C NMR of linolenic acid isolated from *Saussurea involucrata*. Figures S3–S4: ^1^H NMR and ^13^C NMR of linoleic acid isolated from *Saussurea involucrata*.
