# Supplementary material for: Chemical Fingerprint Analysis for Discovering Markers and Identifying Saussurea involucrata by HPLC Coupled with OPLS-DA
Source: J Anal Methods Chem. 2020 Sep 19;2020:7560710. doi: 10.1155/2020/7560710 (PMC7520670; doi:10.1155/2020/7560710)
Supplement: Supplementary Materials — The supplementary data consist of Table S1 and Figures S1–S4. Table S1: ratios of the common peak areas from 72 samples with the filter of median, upper quartile, and 100% of the chromatographic data of each sample. Figures S1–S2: 1H NMR and 13C NMR of linolenic acid isolated from Saussurea involucrata. Figures S3–S4: 1H NMR and 13C NMR of linoleic acid isolated from Saussurea involucrata. [file 7560710.f1.pdf]

Chemical fingerprint analysis for discovering markers and identifying *Saussurea involucrata* by HPLC coupled with OPLS-DA

Qingdong Ma [a], Xiaoxiang Chen[b], Ke Zhang [b,✉], Dahong Yao[c], Lu Yang[d], Hangyu Wang [b], Santai Bulemasi[e], Jian Huang [c], Jinhui Wang[a,b,c,✉]

[a]*School of Pharmacy, Xinjiang Medical University, Urumqi 830054, P.R. China*

[b]*College of Pharmacy （Key Laboratory of Xinjiang Phytomedicine Resource and Utilization, Ministry of Education）, Shihezi University, Shihezi 832002, P.R.China*

[c]*School of Pharmacy, Harbi Medical University, Harbi 150081, P.R.China*

[d] *Economic Forest Product Quality Inspection and Testing Center of the State Forestry Administration (Urumqi), Xinjiang Academy of Forestry, Urumqi 830052, P.R.China;*

[e]*State Forestry Administration of Xinjiang Altai Mountain, Altai 836505, P.R.China*

✉ Ke Zhang, E-mail, tcm_zk@163.com, College of Pharmacy （Key Laboratory of Xinjiang Phytomedicine Resource and Utilization, Ministry of Education）, Shihezi University, Shihezi 832002, China;

✉Jinhui Wang, E-mail, tcm_syphu@163.com, School of Pharmacy, Xinjiang Medical University, Urumqi, 830054, China; School of Pharmacy, Shihezi University (Key Laboratory of Xinjiang Phytomedicine Resource and Utilization, Ministry of Education), Shihezi, 832002, China; School of Pharmacy, Harbi Medical University, Harbi, 150081, China.

Table S1. Ratios of the common peak areas from 72 samples with the median, upper quartile, and 100% of the frequency domain respectively.

Sampels			1	2	3	4	5	6	7	8	9	10
		△				7.82		8.52		12.22		
		□	3.55	5.44	6.28	7.82		8.52	10.51	12.22		13.99
		☆	3.55	5.44	6.28	7.82	8.01	8.52	10.51	12.22	13.54	13.99
S. involucrata	**A**	**1**	**0.023388089**	**0.021545046**	**0.00337579**	**0.031752993**	**0.009406056**	**0.177137081**	**0.016679625**	**0.027582027**	**0.005615361**	**0.100462616**
		**2**	**0.028512101**	**0.008634055**	**0.006011291**	**0.018454568**	**0**	**0.036045459**	**0**	**0.008232943**	**0**	**0.018093296**
		**3**	**0.033158959**	**0.04519278**	**0.002625648**	**0.042712895**	**0.010256945**	**0.129562386**	**0.016321168**	**0.033086317**	**0.003644556**	**0.045278804**
		**4**	**0.093108096**	**0.129921868**	**0.006042789**	**0.086550034**	**0.004674877**	**0.13787225**	**0.07380328**	**0.007782783**	**0.003278217**	**0.018755373**
		**5**	**0.041221278**	**0.104445758**	**0.001461209**	**0.129034342**	**0.008911545**	**0.201477792**	**0.006315933**	**0.017950689**	**0.007736763**	**0.076455044**
		**6**	**0.017523133**	**0.014632367**	**0.001752484**	**0.034533719**	**0.005796054**	**0.231554709**	**0.017444115**	**0.083857413**	**0.005780251**	**0.023435364**
		**7**	**0.003009269**	**0.00188148**	**0.003116768**	**0.013083164**	**0.004024064**	**0.028249906**	**0.003025991**	**0.062625371**	**0.002763692**	**0.021543372**
		**8**	**0.006713999**	**0.004324799**	**0.001443166**	**0.022946215**	**0**	**0.069579397**	**0.001980612**	**0.023296433**	**0**	**0.058912582**
		**9**	**0**	**0.024221166**	**0**	**0.067260001**	**0.025428283**	**0.551953724**	**0.010707755**	**0.061390739**	**0.003894861**	**0.051157048**
		**10**	**0.061940641**	**0.033708672**	**0**	**0.035953152**	**0.017004783**	**0.265587097**	**0.010485583**	**0.055235375**	**0.002622459**	**0.026590341**
		**11**	**0.005409901**	**0.001414104**	**0.001314828**	**0.013076591**	**0**	**0.059173688**	**0**	**0.012674072**	**0**	**0.025479652**
		**12**	**0.086493365**	**0.004422782**	**0.003049377**	**0.011591909**	**0.011314904**	**0.039056287**	**0.003368211**	**0.011118305**	**0**	**0.005749709**
		**13**	**0**	**0.011036924**	**0.002213963**	**0.023466273**	**0.012094571**	**0.127089763**	**0.002297744**	**0.026553703**	**0**	**0.025594377**
		**14**	**0.014210827**	**0.007206944**	**0.002452058**	**0.012411561**	**0.006632855**	**0.084584234**	**0.002587999**	**0.024158747**	**0**	**0.015045556**
		**15**	**0.012802497**	**0.002472416**	**0**	**0.006704412**	**0.002211776**	**0.02224232**	**0**	**0.011135553**	**0**	**0.00741934**
		**16**	**0.027826082**	**0.050200784**	**0**	**0.051953959**	**0.031671979**	**0.36511784**	**0.012138276**	**0.052917641**	**0.005889551**	**0.06979533**
		**17**	**0.072836499**	**0.083798241**	**0.00454531**	**0.061716578**	**0.030143437**	**0.247286097**	**0.018801117**	**0.041808201**	**0.004045759**	**0.007076191**
	**B**	**1**	**0.004114113**	**0.001463717**	**0.004995814**	**0.003378872**	**0**	**0.01883922**	**0**	**0.007469163**	**0**	**0.00841086**
		**2**	**0.014345373**	**0.016099179**	**0.005204518**	**0.02001752**	**0**	**0.061854265**	**0.002399805**	**0.013375977**	**0**	**0.014193466**
		**3**	**0.038828037**	**0.019017018**	**0.003880617**	**0.019594689**	**0**	**0.046222699**	**0.003107061**	**0.016269873**	**0.001133946**	**0.009127674**

Sampels		1	2	3	4	5	6	7	8	9	10
	△				7.82		8.52		12.22		
	□	3.55	5.44	6.28	7.82		8.52	10.51	12.22		13.99
	☆	3.55	5.44	6.28	7.82	8.01	8.52	10.51	12.22	13.54	13.99
	4	0.054343995	0.055323147	0.00577432	0.068547272	0.002556928	0.282611859	0.027527172	0.040178647	0.003489906	0.024108178
	5	0	0.034460175	0.002937802	0.038558913	0.001472427	0.301698558	0.015752476	0.097253157	0.00355871	0.028788637
	6	0.081263473	0.059263445	0.016271571	0.046627085	0.005272181	0.159064246	0.019618015	0.020028039	0.00275391	0.022650067
	7	0.075795398	0.098077435	0.006499522	0.063130059	0.001646094	0.258687278	0.015228921	0.031555647	0.004876682	0.028512718
	8	0.107473803	0.111959191	0.006255094	0.079146321	0	0.103631069	0.009307572	0.006451898	0	0.012294111
	9	0.061724793	0.055248006	0.003262422	0.039901853	0	0.226962147	0.008241872	0.019397749	0.002434116	0.014227442
	10	0.052491705	0.063292685	0.007346021	0.046427235	0	0.229570984	0.011160566	0.084889626	0	0.008950362
	11	0.056833388	0.080703987	0.004916178	0.049227059	0	0.179988438	0.01346514	0.043684119	0.001394033	0.012372594
	12	0.085156281	0.106904233	0.007323649	0.060922683	0.001969237	0.193975678	0.009264382	0.023516827	0.001448215	0.006934245
	13	0.176151887	0.009152135	0.003890208	0.010658933	0	0.042549259	0.003759649	0.033261479	0	0.00134798
	14	0.020950365	0.062188569	0.008927007	0.054790794	0	0.246836159	0.015232324	0.081137672	0.003745722	0.022802177
	15	0.06327741	0.052835688	0.00586766	0.050573594	0	0.161118288	0.011848673	0.0448805	0.001448665	0.027148751
	16	0.058455359	0.04706861	0.006765401	0.047637033	0	0.165099971	0.011821377	0.036539482	0.003162894	0.021077785
	17	0.016162657	0.002959844	0.008552872	0.005688788	0	0.067760538	0	0.017157563	0	0.012044991
C	1	0.026276315	0.03685845	0.005172503	0.023652445	0.027296709	0.129575902	0.008449317	0.060021187	0.003045059	0.024172383
	2	0.039485523	0.016617662	0	0.03520785	0.013185183	0.20238926	0.003957265	0.079566264	0.003561538	0.015410534
	3	0.141758213	0.028490539	0.003669213	0.014516529	0	0.049225507	0.006673576	0.008066342	0	0
	4	0.040595966	0.04553163	0.008461039	0.025112313	0.050284414	0.085817402	0.007057783	0.022580121	0	0.011652305
	5	0.025551011	0.024094331	0	0.016787988	0.010143539	0.010016386	0.00160136	0.005998487	0	0.003089589
	6	0.024231532	0.031258137	0	0.026337511	0.022800378	0.038197023	0	0.022835605	0	0.005113335
D	1	0.263771263	0.144807124	0	0.146514973	0	0.029482088	0	0.011286573	0	0
	2	0.118097013	0.024887957	0.008864034	0.067230572	0	0.105666239	0	0.028959571	0	0.014750087

Sampels		1	2	3	4	5	6	7	8	9	10
	△				7.82		8.52		12.22		
	□	3.55	5.44	6.28	7.82		8.52	10.51	12.22		13.99
	☆	3.55	5.44	6.28	7.82	8.01	8.52	10.51	12.22	13.54	13.99
	3	0.25140754	0	0	0.072496927	0	0.080394341	0	0.039337455	0	0.018140344
S. orgaadayi	1	0.008687217	0.005750048	0.015434375	0.013371181	0.001460484	0.039831399	0.007345701	0.036085061	0.006563845	0.011948887
	2	0	0	0.007532445	0.015458905	0	0.032160275	0	0.003519195	0.016456522	0.021080307
	3	0.032756899	0.028039657	0	0.031748014	0.007226559	0.303271247	0.00778087	0.007412365	0	0.012826847
	4	0	0.069438211	0.008411612	0.071842673	0.012640795	0.104946781	0.025915432	0.024343181	0.002556077	0.04437703
	5	0	0.08841345	0	0.066042915	0.005045748	0.032276253	0.006228694	0.007675909	0.001119316	0.007303887
	6	0.003965244	0.038492978	0.002227137	0.048980631	0.005237816	0.147895366	0.02594484	0.051756357	0.00162063	0.019591815
	7	0.007349336	0.050637148	0	0.041559157	0.016372732	0.089335963	0.021729625	0.028419235	0.00249859	0.013550657
	8	0.002556856	0.010951617	0.006747443	0.016162743	0	0.055036229	0.010329964	0.102908955	0.003063151	0.020686844
S. laniceps	1	0.04161977	0	0.024100547	0	0	0.045860639	0	0.018744675	0.066160357	0.069280963
	2	0.005037797	0	0	0.003571385	0.005914987	0.026818593	0	0.01076966	0.016344492	0.046158057
	3	0.011052784	0.008247652	0	0.005729622	0.016879183	0.072765238	0.004753103	0.003055369	0.027948557	0.072734822
	4	0.01312736	0.006851842	0	0.011986995	0	0.031163554	0.006118499	0.026405599	0.003773294	0.010979964
	5	0.030033043	0.007839588	0	0.011759952	0	0.029652633	0.001543868	0.02195723	0.022527418	0.014618302
S. medusa	1	0.007073478	0	0	0.004289609	0.009036109	0.06215014	0.006950501	0.016510618	0.112485722	0.063595851
	2	0	0.015496124	0	0.016154161	0	0.164383429	0	0	0.463196705	0
	3	0.011302224	0	0	0	0	0.044962061	0	0.102982676	0.118151201	0.029760308
	4	0	0	0	0	0.001951102	0.029559233	0	0.00924889	0.052435278	0.036056342
	5	0	0	0	0	0	0.042586515	0	0.094129993	0.146933366	0.029358527
	6	0	0.004251039	0	0.003990749	0	0.039845	0	0.093326146	0.054564691	0.031762429
S. tridactyla	1	0.021480504	0.016497082	0	0.01720697	0.004528834	0.046585942	0	0.066759505	0.257289543	0.009429861
	2	0.007068444	0.009076388	0	0.013920139	0.007188861	0.048993228	0.003735317	0.096362641	0.255506192	0.023627509

		1	2	3	4	5	6	7	8	9	10
	△				7.82		8.52		12.22		
**Sampels**	□	3.55	5.44	6.28	7.82		8.52	10.51	12.22		13.99
	☆	3.55	5.44	6.28	7.82	8.01	8.52	10.51	12.22	13.54	13.99
	3	0.006772291	0.011115766	0.00647826	0.00795267	0.008288174	0.071764379	0.003242852	0.094689784	0.241070061	0.038588288
	4	0.024743307	0	0	0.033147528	0.002743469	0.032970081	0	0.07021949	0.287573719	0.005475432
	5	0.02817971	0.003712609	0.002218653	0.003349087	0.007111886	0.037525728	0	0.064505114	0.291943334	0.016469161
S. simpsoniana	1	0.002040695	0.01034157	0.021952737	0.010028262	0.024127137	0.112296893	0.019051941	0.220276518	0.14330045	0.080121125
	2	0.015447048	0.016274883	0.015379042	0.022243016	0.005390087	0.118672141	0.00620419	0.126093891	0.148527845	0.088759548
	3	0	0.006466746	0.013467092	0.006570877	0.034114555	0.105347008	0.023306053	0.193160498	0.13185643	0.09729005
	4	0.01531081	0.014513279	0	0.02020879	0.013185934	0.07432059	0.010889757	0.15302947	0.148357082	0.040287935
	5	0	0.00846599	0	0.014047661	0.038614988	0.095921467	0.013525641	0.181001181	0.146451164	0.10237317
**Total**	72										

Note: Note: the letter A is the master haplotype of *S. involucrata*, which possessed another three haplotypes with three variable sites at the 103rd (T-C), 156th (C-T), and 167th (T-C) for D, C, and B, respectively. ☆, □, △: Retention times (min) in a common pattern from the median, upper quartile, and 100% of the frequency domain were showed.

Table S1. Continuing.

Sampels			11	12	13	14	15	16	17	18	19	20
		△					19.21		20.87		38.98	40.78
		□	14.15				19.21	20.42	20.87		38.98	40.78
		☆	14.15	14.69	16.99	18.18	19.21	20.42	20.87	31.95	38.98	40.78
S. involucrata	**A**	**1**	**0.017183944**	**0.010961422**	**0.229836794**	**0.018865124**	**0.034167856**	**0**	**0.076523205**	**0.089422384**	**0.049820113**	**0.056274474**
		**2**	**0.002724056**	**0**	**0.352697045**	**0.016178218**	**0.038209713**	**0**	**0.067141835**	**0.087252174**	**0.096182**	**0.215631246**
		**3**	**0.002507604**	**0.006394461**	**0.221192906**	**0.008606715**	**0.06376348**	**0.003494969**	**0.112376197**	**0.037569608**	**0.086714241**	**0.095539362**
		**4**	**0**	**0.019493026**	**0.001438424**	**0.00638992**	**0.052444968**	**0**	**0.151937017**	**0.033732443**	**0.060987642**	**0.111786992**
		**5**	**0**	**0.026802472**	**0.005902445**	**0.005178839**	**0.030919054**	**0.014486644**	**0.10248061**	**0.056152638**	**0.080639465**	**0.082427481**
		**6**	**0.040692827**	**0.018649028**	**0.217843219**	**0.016771397**	**0.019630556**	**0.01279275**	**0.043702333**	**0.094513695**	**0.048716753**	**0.050377833**
		**7**	**0.015100571**	**0**	**0.654144809**	**0.075138788**	**0.004937093**	**0.026009378**	**0.006461196**	**0.037720371**	**0.010820658**	**0.02634406**
		**8**	**0.00165117**	**0.003488331**	**0.636040962**	**0.070627083**	**0.003737392**	**0.006922992**	**0.00474328**	**0.02092603**	**0.014337675**	**0.048327881**
		**9**	**0.023493646**	**0**	**0.002343694**	**0.002348535**	**0.019098442**	**0.003711944**	**0.036648483**	**0.027099089**	**0.0541432**	**0.035099391**
		**10**	**0.006909406**	**0.017236036**	**0.022160921**	**0.007373506**	**0.030999559**	**0.015371129**	**0.075356488**	**0.124143368**	**0.092137992**	**0.099183492**
		**11**	**0.001596668**	**0.002105168**	**0.658871992**	**0.054735661**	**0.009166146**	**0.008511181**	**0.018359366**	**0.03769628**	**0.020588565**	**0.069826138**
		**12**	**0.007308094**	**0**	**0.216793929**	**0.014094712**	**0.023843795**	**0.01228628**	**0.081661583**	**0.168373391**	**0.107702361**	**0.191771004**
		**13**	**0.007329791**	**0.005182646**	**0.572156486**	**0.023476659**	**0.014632579**	**0.007050406**	**0.029507154**	**0.041359037**	**0.030631272**	**0.038326653**
		**14**	**0.016740566**	**0.004164103**	**0.564312596**	**0.034988072**	**0.007546968**	**0.048676938**	**0.031493796**	**0.043925459**	**0.032811645**	**0.046049077**
		**15**	**0.024684641**	**0**	**0.552722536**	**0.044516042**	**0.006609943**	**0**	**0.017641083**	**0.002960985**	**0.027643794**	**0.258232662**
		**16**	**0.016794274**	**0.00432872**	**0**	**0**	**0.037355448**	**0**	**0.080146083**	**0.018334007**	**0.100137585**	**0.07539244**
		**17**	**0.020943983**	**0**	**0**	**0**	**0.046354187**	**0.005601872**	**0.109585643**	**0.084570213**	**0.088868114**	**0.07201856**

Sampels		11	12	13	14	15	16	17	18	19	20
	△					19.21		20.87		38.98	40.78
	□	14.15				19.21	20.42	20.87		38.98	40.78
	☆	14.15	14.69	16.99	18.18	19.21	20.42	20.87	31.95	38.98	40.78
B	1	0.005647825	0	0.344195583	0.015455054	0.004760537	0.021010238	0.011116251	0.081974045	0.037400518	0.42976819
	2	0.003029661	0	0.349417515	0.006039475	0.008675344	0.032668632	0.014393009	0.12555602	0.031018303	0.281711939
	3	0.004521522	0	0.281014742	0.008608008	0.019745882	0.038947374	0.081963643	0.144031638	0.078651189	0.185334388
	4	0.0140462	0.006467354	0	0	0.04686162	0.020045622	0.079885651	0.091454493	0.077856847	0.098920788
	5	0.042891995	0.008828753	0.002287446	0.003705538	0.038273968	0.034817291	0.064143881	0.120746035	0.069038974	0.090785264
	6	0	0.003264565	0	0	0.037397799	0.031501206	0.112282776	0.06254051	0.156527223	0.163673889
	7	0.008172542	0.017322541	0.00154125	0	0.035416115	0.03026488	0.070930955	0.099177279	0.066454476	0.086710206
	8	0.00364796	0.006966731	0	0.006160751	0.03446135	0.041535612	0.083814816	0.22474465	0.063483731	0.09866534
	9	0.017625112	0.009748477	0	0.007085058	0.035321624	0.063061136	0.070153403	0.237007552	0.06006921	0.068528028
	10	0.063205715	0.003559908	0	0.002823173	0.028409435	0.045124779	0.08792646	0.090307677	0.079209829	0.09530384
	11	0.017240092	0	0	0	0.042782423	0.056384753	0.090080516	0.199398685	0.066234812	0.085293784
	12	0.011970092	0.002564032	0	0	0.043846324	0.02324437	0.112280022	0.119294747	0.093500597	0.095884388
	13	0.039523228	0	0	0	0.02968156	0.048372985	0.090113456	0.238083235	0.107593678	0.165860328
	14	0.03742565	0	0.004057252	0	0.039119592	0.033436282	0.065485941	0.167397691	0.063330493	0.073136308
	15	0.054512421	0	0	0.002832945	0.041129387	0.02430175	0.132190331	0.154229535	0.076954258	0.094850144
	16	0.034152353	0	0.007396983	0.002689623	0.038353201	0.018086499	0.149583842	0.182013058	0.081182736	0.088913792
	17	0.007702228	0	0.34845286	0.009231896	0.017138908	0.052361885	0.028484564	0.197396332	0.031586182	0.177317892
C	1	0.050857124	0.009658474	0.075005995	0.008748919	0.035301997	0	0.137549617	0.057963606	0.156897466	0.123496531
	2	0.108572771	0.00418037	0	0.004151464	0.032165805	0.006570607	0.11263711	0.075304062	0.106510678	0.140526056
	3	0	0.006493497	0	0	0.122933456	0	0.33342426	0.006061914	0.119476995	0.15920996
	4	0.008854232	0.004318091	0	0.008788817	0.049503655	0	0.169742694	0	0.239418074	0.222281463
	5	0.015901424	0	0	0.005035759	0.015300077	0	0.672840745	0	0.085694582	0.107944721

Sampels			11	12	13	14	15	16	17	18	19	20
		△					19.21		20.87		38.98	40.78
		□	14.15				19.21	20.42	20.87		38.98	40.78
		☆	14.15	14.69	16.99	18.18	19.21	20.42	20.87	31.95	38.98	40.78
		6	0.02517159	0.003273281	0	0.016590884	0.059173923	0	0.15394296	0.018883355	0.237991467	0.31419902
	D	1	0.007653009	0	0.016303033	0	0.022222345	0	0.095580456	0.001600762	0.075968812	0.184809561
		2	0.011882403	0.022613694	0.134608723	0	0.064603674	0	0.15739019	0.002176395	0.112423783	0.125845664
		3	0.016195338	0.010938068	0	0	0.06321384	0	0.212120724	0	0.133632258	0.102123164
S. orgaadayi		1	0.058430464	0.002880232	0.661676256	0.010026187	0.040517664	0.027266608	0.003219777	0.002132862	0.005801431	0.041570323
		2	0.018118755	0.002369638	0.608932912	0.016672448	0.095193589	0.062050753	0.005023347	0.043186162	0.004067924	0.048176823
		3	0.020470071	0.001388265	0.005200711	0.015229589	0.023416872	0	0.051459946	0.335007765	0.053285384	0.06347894
		4	0.102105508	0.008069644	0.016788493	0.026475806	0.04437703	0	0.136832628	0.003816417	0.148768782	0.1482939
		5	0.036692676	0	0.004342576	0.015961172	0.032692861	0	0.106761878	0.429502578	0.077047513	0.082892573
		6	0.071820811	0.00554107	0.003862521	0.019328996	0.042097596	0	0.065718585	0.241712164	0.095435789	0.108769654
		7	0.058938571	0.004394091	0.010669905	0.019615236	0.046527255	0	0.066420056	0.352692605	0.070762103	0.098527735
		8	0.140950836	0	0.502622866	0.005172416	0.002443804	0.002532759	0.004421561	0.01795772	0.01875113	0.076703107
S. laniceps		1	0.015669651	0.078186963	0.104563098	0.009863219	0	0.168987837	0.019637028	0	0.139112415	0.198212839
		2	0.112161779	0.00432333	0.523937005	0.0418371	0	0	0.005505094	0	0.021726713	0.175894008
		3	0.010872135	0	0.733673249	0	0	0	0.007914464	0	0.009851375	0.014522448
		4	0.011101896	0	0.768466353	0.008006681	0.003718468	0	0.006391309	0	0.051868204	0.040039983
		5	0.013643482	0	0.757615901	0.007264841	0.002457423	0.002306113	0.006433922	0	0.039719972	0.030626312
S. medusa		1	0	0.006926322	0.597046465	0.011255117	0	0.016301348	0	0	0	0.08637872
		2	0.160622296	0.013996187	0	0	0	0.017279921	0.039327383	0	0.028182689	0.081361104
		3	0.027403503	0.013469156	0.382134796	0.065145621	0	0.037367118	0	0	0.027721541	0.139599794
		4	0.003128011	0.009477752	0.747579798	0.01530729	0	0.016884199	0	0	0	0.078372103
		5	0.009518668	0.005785084	0.570059143	0.015172121	0	0.010361227	0	0	0.011705438	0.064389918

		11	12	13	14	15	16	17	18	19	20
**Sampels**	△					**19.21**		**20.87**		**38.98**	**40.78**
	□	**14.15**				**19.21**	**20.42**	**20.87**		**38.98**	**40.78**
	☆	**14.15**	**14.69**	**16.99**	**18.18**	**19.21**	**20.42**	**20.87**	**31.95**	**38.98**	**40.78**
	**6**	**0.019686391**	**0.009196003**	**0.658443717**	**0.016603125**	**0**	**0.007703276**	**0**	**0**	**0.013936105**	**0.04669133**
S. tridactyla	**1**	**0.146940724**	**0.004596616**	**0.196985188**	**0.044701025**	**0**	**0.075248126**	**0.008521091**	**0**	**0.04963087**	**0.033598117**
	**2**	**0.156274629**	**0.020709217**	**0.134903122**	**0.024167576**	**0**	**0.078881187**	**0.020140851**	**0**	**0.051049941**	**0.048394759**
	**3**	**0.174472764**	**0.014318741**	**0.10806688**	**0.079914311**	**0**	**0.080359877**	**0.003754881**	**0**	**0.02431421**	**0.02483581**
	**4**	**0.10110199**	**0.010053937**	**0.092085485**	**0.112159443**	**0**	**0.168312748**	**0.008980774**	**0**	**0.022409844**	**0.028022752**
	**5**	**0.105074102**	**0.009080061**	**0.088754571**	**0.103396275**	**0.00312347**	**0.172762725**	**0.016880996**	**0**	**0.021194001**	**0.024718517**
S. simpsoniana	**1**	**0.004504855**	**0.100089443**	**0.027662133**	**0.011099098**	**0.007088352**	**0.095395006**	**0.030570912**	**0**	**0.034244402**	**0.045808473**
	**2**	**0.00279934**	**0.110281314**	**0.030639075**	**0.021942223**	**0.00236319**	**0.116940618**	**0.016643682**	**0**	**0.059396229**	**0.076002639**
	**3**	**0.016779299**	**0.071400711**	**0.040704847**	**0.020940443**	**0**	**0.102427795**	**0.01380302**	**0**	**0.019644976**	**0.102719598**
	**4**	**0.005150251**	**0.122335773**	**0.027590629**	**0.027017755**	**0.012141281**	**0.145598955**	**0.062338908**	**0**	**0.051617644**	**0.056105158**
	**5**	**0.012385375**	**0.069087568**	**0.056246984**	**0.020573155**	**0.00189712**	**0.104843165**	**0.020099996**	**0**	**0.021599245**	**0.092866132**
**Total**	**72**										

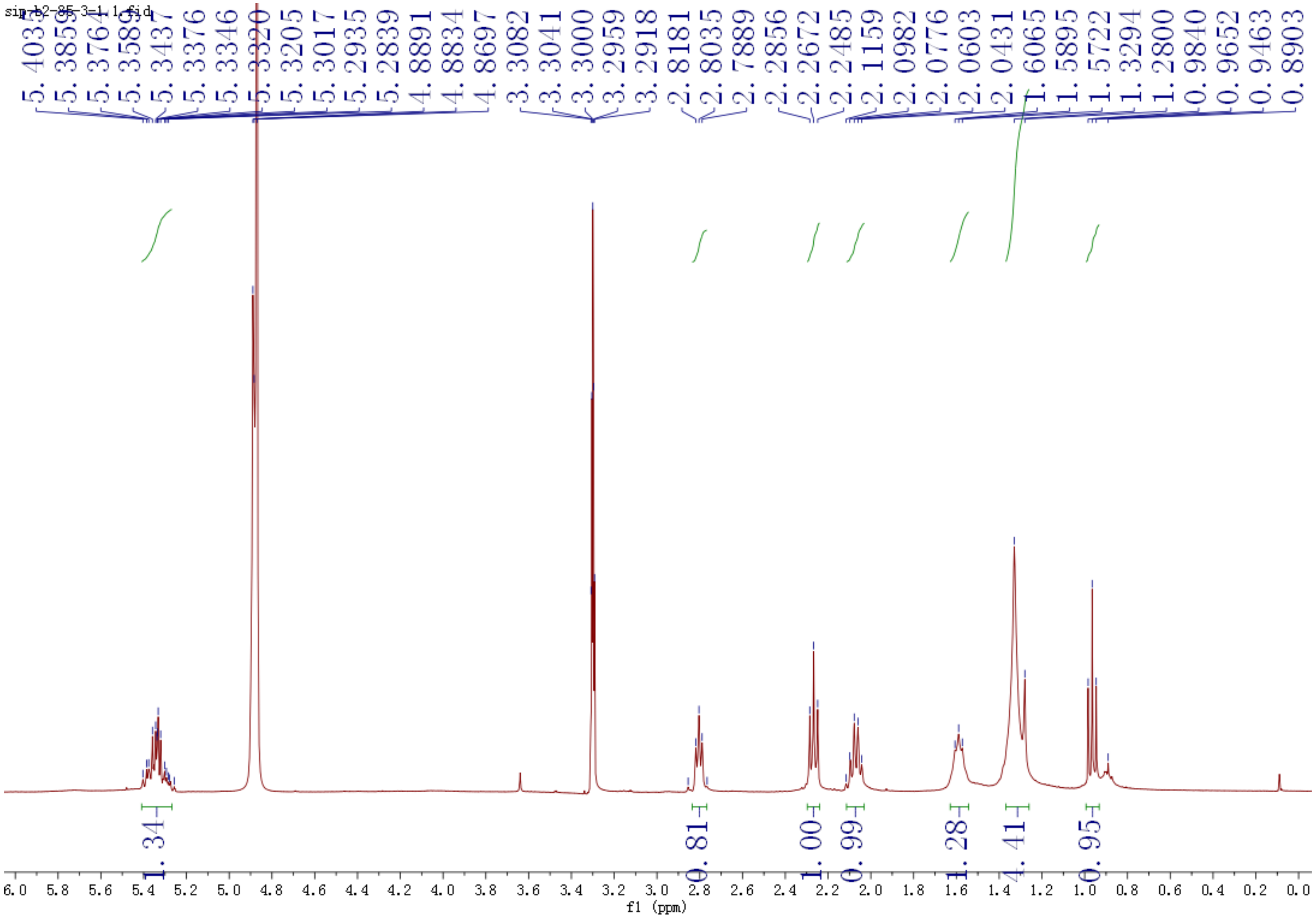

Figure S1, ^{I}H NMR of linolenic isolated from *Saussurea involucrata* (400MHz, CD$_3$OD).

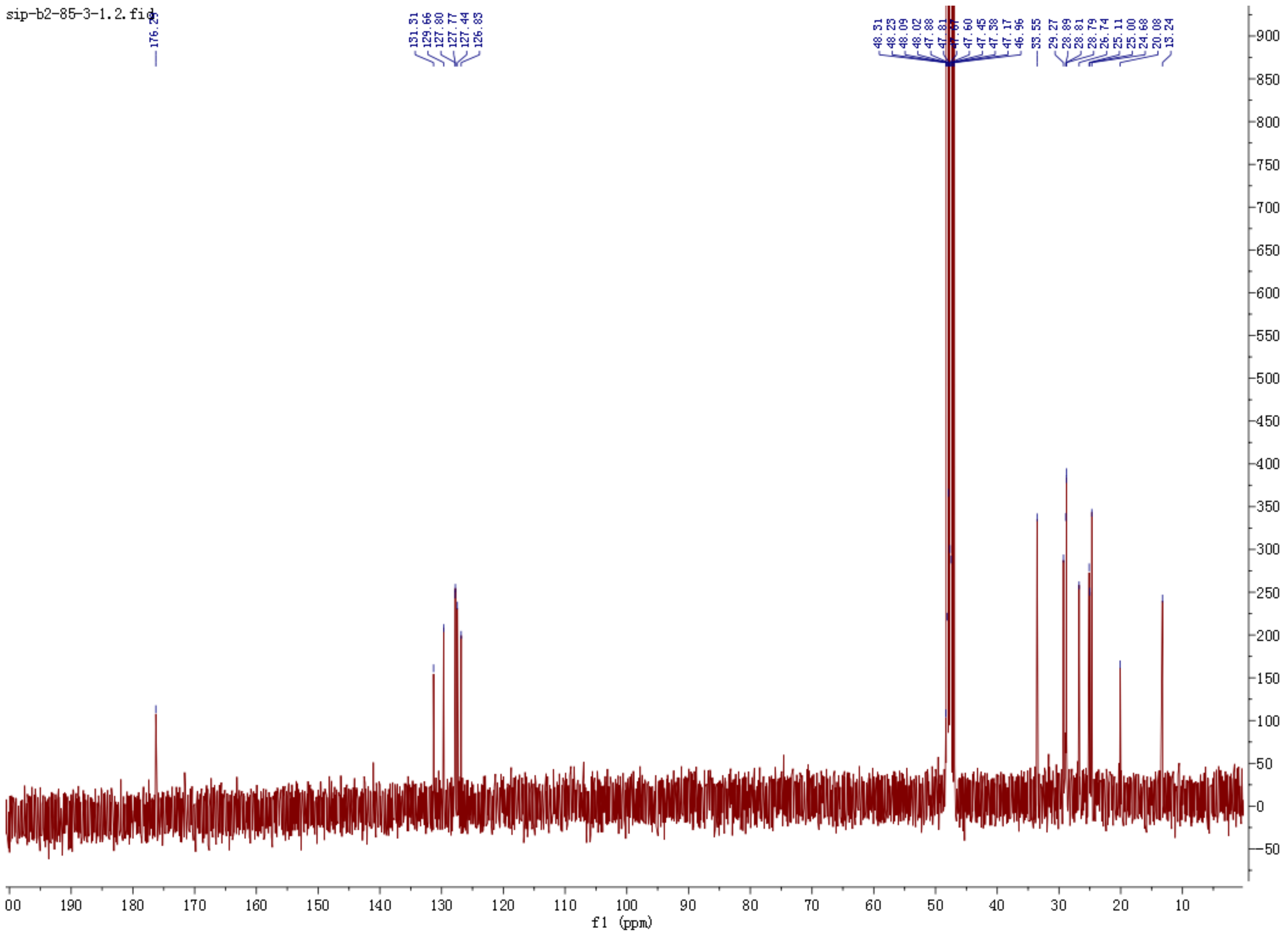

Figure S2, ^{13}C NMR of linolenic isolated from *Saussurea involucrata* (100MHz, CD_3OD).

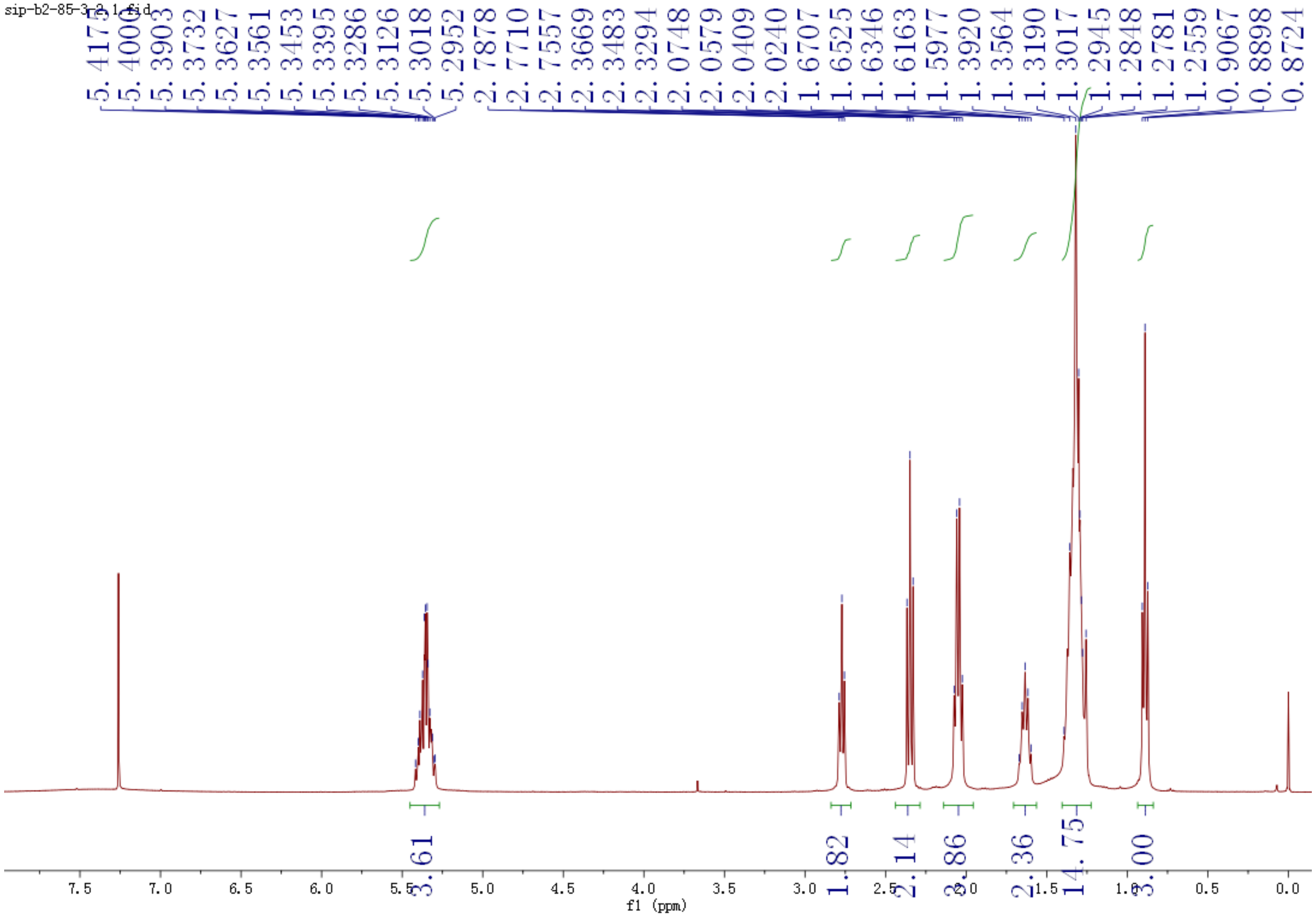

Figure S3: ^{1}H NMR of linoleic isolated from *Saussurea involucrata* (400MHz, $CDCl_3$).

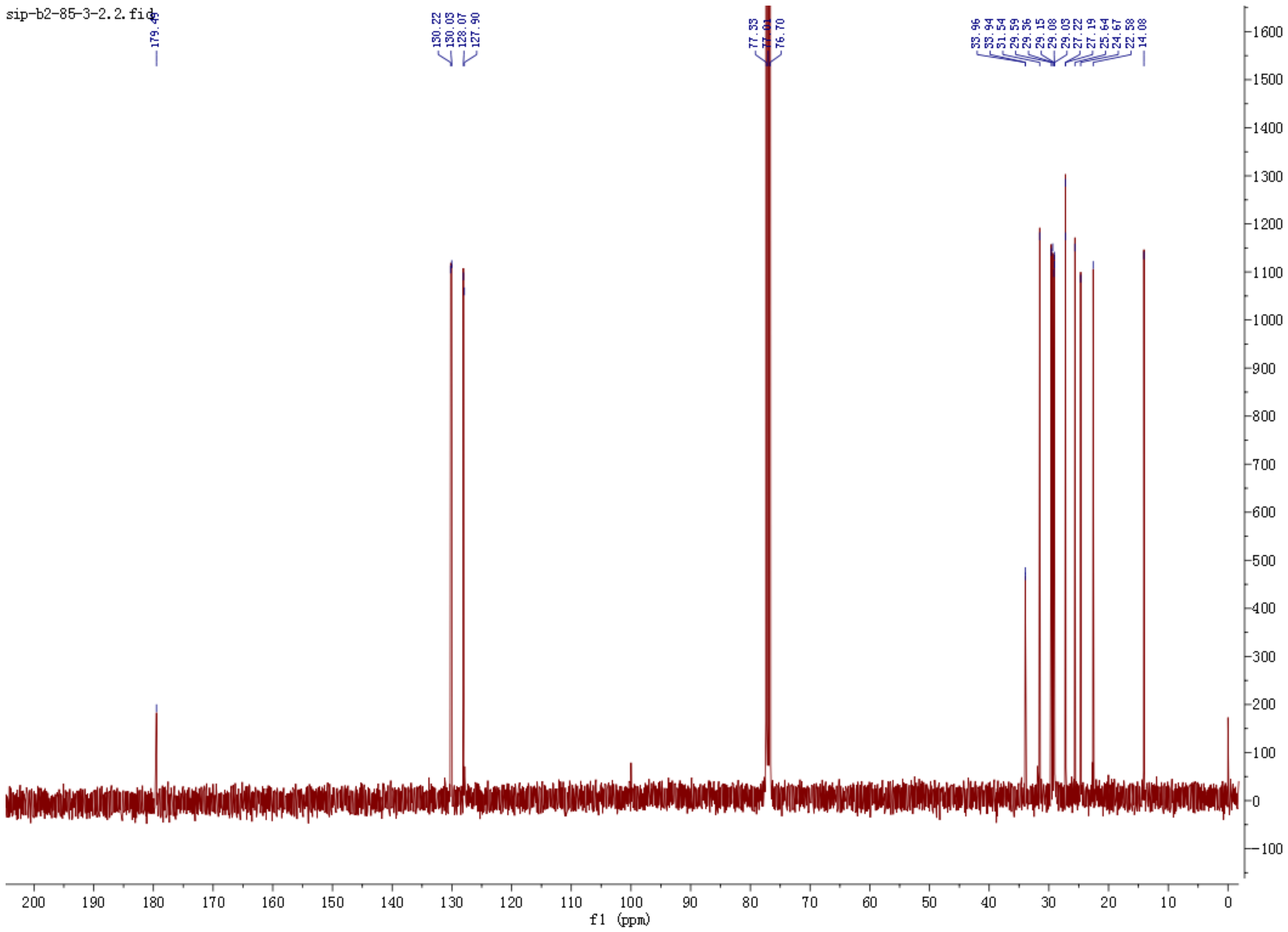

Figure S4: ^{13}C NMR of linoleic isolated from *Saussurea involucrate* (100MHz, $CDCl_3$).
